# Vitamin D Status in Spanish Elite Team Sport Players

**DOI:** 10.3390/nu13041311

**Published:** 2021-04-15

**Authors:** Jara Valtueña, Raquel Aparicio-Ugarriza, Daniel Medina, Antonia Lizarraga, Gil Rodas, Marcela González-Gross, Franchek Drobnic

**Affiliations:** 1 ImFine Research Group, Universidad Politécnica de Madrid, 28040 Madrid, Spain; jara.valtuena@gmail.com (J.V.); apariciougarriza.raquel@gmail.com (R.A.-U.); 2 Medical Services FC Barcelona, 08970 Barcelona, Spain; dmedina@monumentalsports.com (D.M.); mlizarraga@ub.edu (A.L.); gil.rodas@fcbarcelona.cat (G.R.); docdrobnic@gmail.com (F.D.); 3 CIBER Physiopathology of Obesity and Nutrition (CIBEROBN), Instituto de Salud Carlos III (ISCIII), 28029 Madrid, Spain; 4 GIRSANE-CAR (Centre d’Alt Rendiment Sant Cugat del Vallès), 08173 Sant Cugat del Vallès, Spain

**Keywords:** elite players, 25(OH)D, vitamin D deficiency, supplementation

## Abstract

Low vitamin D is usual; however, data are limited for elite team players. The aim was to investigate the vitamin D levels in Football Club Barcelona (FCB) first division players of six sport modalities. Ninety-five elite male players (27.3 ± 4.6 y) belonging to FCB provided data for vitamin D throughout a season. In this study, 25(OH)D was measured in serum by chemiluminescent immunoassay. Outdoor/indoor training and supplementation were also considered. Total mean 25(OH)D concentrations were 91.9 ± 23.1 nmol/L in all players, with higher mean levels among supplemented players (94.7 ± 24.3 nmol/L). Around 25% of the team players were below optimal levels (<75 nmol/L), but none were below 50 nmol/L. Caucasian, supplemented football and handball players had the highest mean vitamin D concentrations over the whole year, whereas basketball players (indoor training) had the lowest ones. The highest rate of vitamin D insufficiency was found in spring (40%). A positive significant effect was observed for the interaction between indoor/outdoor training and supplementation with 25(OH)D concentrations (*p* < 0.05). Those team players training outdoors with supplementation had higher total vitamin D concentrations than those with indoors training and/or supplementation. A positive interaction of outdoor training with supplementation exists to determine 25(OH)D concentrations in team players.

## 1. Introduction

The widespread prevalence of low vitamin D status with insufficient blood concentrations (<75 nmol/L) is well documented [1,2,3,4]. Even if there is a paucity of research investigating the prevalence of vitamin D deficiency in elite sport, especially for some sport modalities [5], available data seem to indicate that vitamin D insufficiency is also affecting athletes and team players worldwide [6]. The available data show that vitamin D insufficiency varies from 16% to 100%, and it is different across latitude, season, sex, type of sport, and socio-cultural and racial factors [6,7,8,9], and which may impact on health and training ability [10,11,12].

Vitamin D is fundamental for a huge quantity of physiological processes; for example: immune system, protein synthesis, inflammatory response, or cellular growth [13,14]. There is no strong data that players have an upper daily requirement more than the general population [15,16], but the finding of vitamin D muscle receptors implied a noteworthy role for vitamin D in muscle tissue [13,17,18], indicating that players could be an at-risk population. Superior physiological and performance benefits have been observed with >75 nmol/L because 25(OH)D levels >100 nmol/L of vitamin D are stored in muscle and fat for use later [19].

A connection between serum 25(OH)D levels with muscle functions and bone mass [17,20,21,22,23,24] has been observed before. Insufficient vitamin D levels produce atrophic changes in type II muscle fibers, which are key in elite players [5]. A recent review found that optimal serum 25(OH)D levels are essential to avoid injuries and to obtain benefits for their physical performance [6,24]. Testosterone and vitamin D are together essential for muscle outcomes, and both improve physical fitness [21]. Deficiencies of vitamin D have a negative effect on physical performance and health, increasing the risk of stress fractures [25,26].

Reports have shown vitamin D deficiency among Spanish athletes [11], and soccer [27] and basketball players [28], although Spain is a Mediterranean country. However, very few data are available in first division players, as research usually in elite sport is very restricted. Some studies have found vitamin D insufficiency in team players considering only outdoor training [29], but there is a lack of vitamin D levels, supplementation, and reliable tests [30] and how these affect vitamin D concentrations throughout the season.

Therefore, the main aims of this study were (1) to investigate, for the first time, vitamin D concentrations in Barcelona Football Club (FCB) first division players of six sport modalities during one competition season, and (2) to analyze how training environment (outdoor or indoor), skin color, sport modality, season, and supplementation could affect vitamin D concentrations during the season. Results should contribute to diagnosing the necessity of optimal vitamin D status in team players across the season.

## 2. Materials and Methods

This longitudinal study included 95 male team players belonging to FCB, from the first division football team (n = 22), second division football team (n = 21), indoor football (12), basketball (n = 12), handball (n = 17), and roller hockey (n = 11). The study took place during the season 2011–2012. They were assessed for vitamin D status using subjects as the primary sampling unit and sport as the secondary sampling unit. Inclusion criteria was active sport elite players, and no declared health problems.

Data were obtained within their routine medical assessments. All players signed an informed consent in order that data obtained could be used for research anonymously.

### 2.1. Biochemical Analyses

Fasting blood samples (12 h) were collected by venipuncture between 8 a.m. to 9 a.m. A total of 20 mL of whole blood was collected from each player. Blood was collected in EDTA tubes, instantaneously located on ice, and centrifuged within 30 min (3500 rpm for 15 min). The supernatant fluid was transported at a stable temperature of 4–7 °C to the central laboratory in Barcelona and deposited there at −80 °C pending examination. Plasma 25(OH)D was analyzed by chemiluminescent immunoassay in a Roche Elecsys Analyzer 170 [31]. The sensitivity was 4.01 ng/mL 25(OH)D and CV was 8.5%.

### 2.2. Seasonality

The date of the collected blood sample was recorded for each player. Blood samples were obtained from the same subjects during the sport’s season, on three occasions, and seasonality was categorized as autumn (A: 21 September to 20 December), winter (W: 21 December to 20 March), and spring (S: 21 March to 20 June) following a previous study [32].

### 2.3. Vitamin Supplementation

Vitamin supplementation was obtained through clinical anamnesis of the team players. Players took 1000 IU for 5 days (from Monday to Friday) during the training and competition season. They were organized into two groups: supplement (1000 IU) and non-supplement consumers.

### 2.4. Statistical Analysis

Outcomes showed a normal distribution, and residuals displayed a satisfactory pattern. Descriptive values are shown as mean ± SD, minimum, and maximum. Frequency is disclosed as a valid percentage. Vitamin D status was classified into two groups (vitamin D sufficiency/optimal levels ≥75 nmol/L and vitamin D insufficiency < 75 nmol/L) following international guidelines [33]. The Pearson correlation coefficient was used to assess the association between 25(OH)D concentrations with age and anthropometric characteristics of the subjects (weight and height). The differences between vitamin D concentrations and sport modality, season, or supplementation were analyzed through one-way ANOVA. Linear regression was computed with 25(OH)D as the dependent variable to analyze the association with environment training, supplementation, sport modality, and anthropometric variables. One-way analysis of covariance (ANCOVA) was performed to analyze the influence of the interaction between training environment and supplementation on vitamin D concentrations and it is represented as a figure. The dichotomous variables of indoor/outdoor training and supplementation (yes/no) were entered as fixed factors and 25(OH)D concentrations were entered as dependent variables controlled by age, height, and weight. Analyses were performed using Statistical Package for Social Sciences software (SPSS, version 20.0 for IBM; SPSS, Chicago, IL, USA), and values of *p* < 0.05 were considered statistically significant.

## 3. Results

Descriptive characteristics are shown in Table 1. Most players were Caucasian (87.4%), and age range was from 18.8 to 37 years. Caucasian players presented significantly higher vitamin D levels than Black team players (*p* < 0.05). Total mean 25(OH)D concentrations of all players during the whole year was 91.9 ± 23.1 nmol/L, with the highest mean levels among outdoor training (99.5 ± 21.0 nmol/L) and supplemented players (94.7 ± 24.3 nmol/L) independently. Almost half of the players, those including handball, basketball, roller hockey, and indoor football players, had indoor training during the whole year, whereas football first and second division, representing 45% of the sample, had outdoor training. Approximately 42% of the team players took vitamin D supplements from October to June.

**Figure 1:**
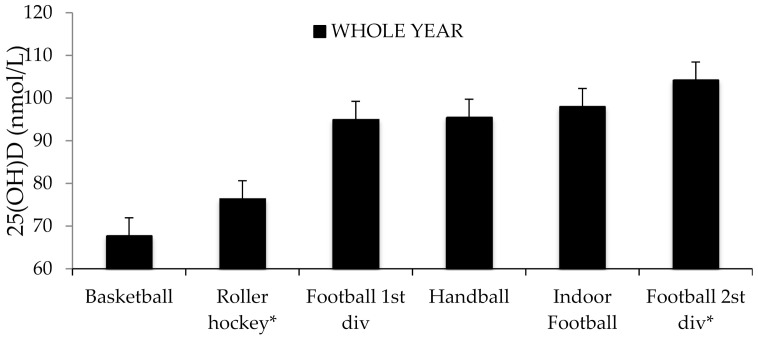
Mean 25(OH)D concentrations of the whole year according to sport modality as mean ± standard error. * Football 2nd division, indoor football, and roller hockey (indoor) were fully supplemented during the whole year.

Figure 1 represents mean 25(OH)D concentrations during the whole year in each sport. Considering vitamin D groups, around 25% of the team players were below optimal levels (<75 nmol/L), but none were below 50 nmol/L.

**Figure 2:**
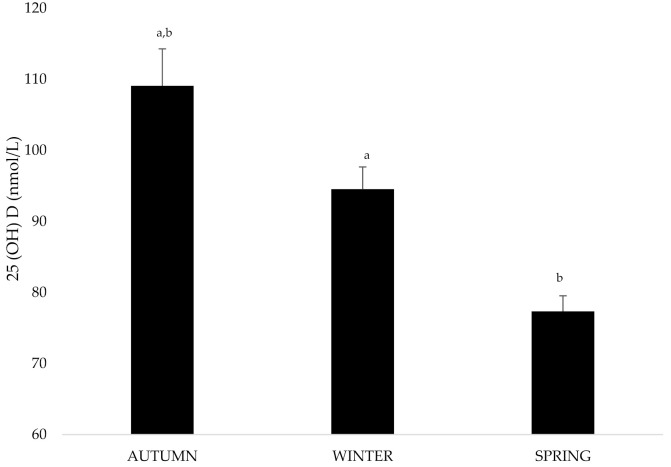
Differences in mean 25(OH)D concentrations (nmol/L) by season. Significant differences were set up as follows (^a^ *p* < 0.05 and ^b^ *p* < 0.01).

Significantly higher vitamin D concentrations were observed in autumn when compared with winter and spring (*p* < 0.05 and 0.01, respectively) (Figure 2).

The highest rate of vitamin D insufficiency (<75 nmol/L) was found in spring (40% of the team players), followed by winter (35%), and autumn (10%). Spring vitamin D concentrations were significantly and positively related to supplementation. In fact, supplemented players had significantly higher vitamin D concentrations during springtime (Table 2). Although not significant, supplemented players presented also lower weight.

**Figure 3:**
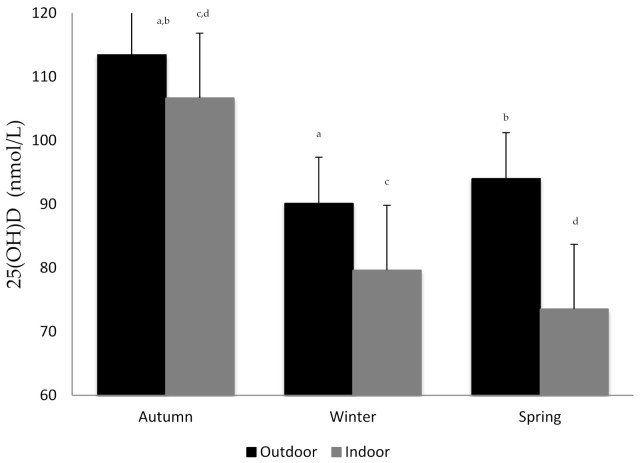
Mean 25(OH)D concentrations according to indoor/outdoor training by season. ^a,b,c,d^ Statistically significant differences (*p* < 0.05) between autumn and the other seasons.

Figure 3 displays mean vitamin D concentrations according to indoor/outdoor training by season.

Table 3 shows descriptive values of vitamin D concentrations according to sport type (training environment) and supplementation by season. Differences in mean 25(OH)D concentrations were observed by sport modality and supplementation throughout the year. Roller hockey, indoor football, and football second division were fully supplemented throughout the year. On the contrary, only 5.9% of handball players, 27.3% of the football first division players, and around 60% of the basketball players were supplemented (Table 3). In general, those team players that took supplements had higher vitamin D levels than those without supplementation (*p* < 0.05 for football players).

**Figure 4:**
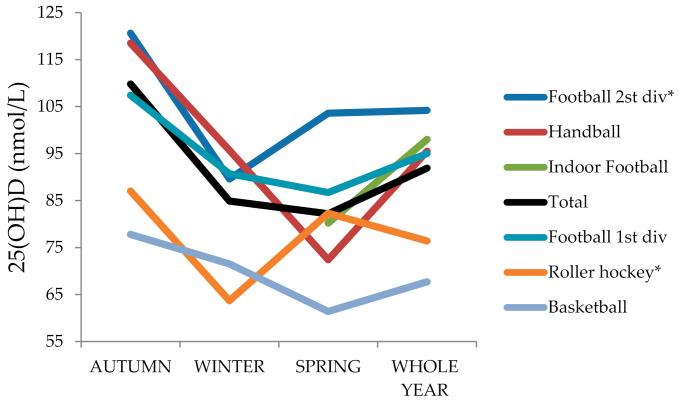
Mean 25(OH)D concentrations according to sport modality by season. * Football 2nd division, indoor football, and roller hockey (indoor) were fully supplemented during the whole year (100% of the players).

The highest vitamin D concentrations were found in autumn, especially in supplemented players, whereas the lowest were observed during spring for most of the sport modalities, and particularly in non-supplemented players (*p* < 0.05). The highest mean vitamin D concentrations for the whole year were observed in outdoor football players, especially second division fully supplemented during the year, whereas the lowest concentrations were observed in basketball players (*p* < 0.001). Big seasonal differences were observed in handball and indoor football players, with high values at the beginning of the season in October but with a progressive decrease in the following months, especially in non-supplemented handball players, with insufficiency mean values in spring (*p* < 0.01). On the contrary, indoor football players, fully supplemented during the entire season, had optimal vitamin D mean status during springtime. Basketball and roller hockey players’ mean vitamin D concentrations were at insufficiency levels during winter, but roller hockey, fully supplemented, showed a recovery in spring. Minimal vitamin D changes throughout the year were observed in basketball players whose vitamin D concentration remained low and quite stable throughout the year, with insufficiency levels in winter and spring, and surprisingly, no positive influence was found regarding supplementation (Figure 4).

**Figure 5:**
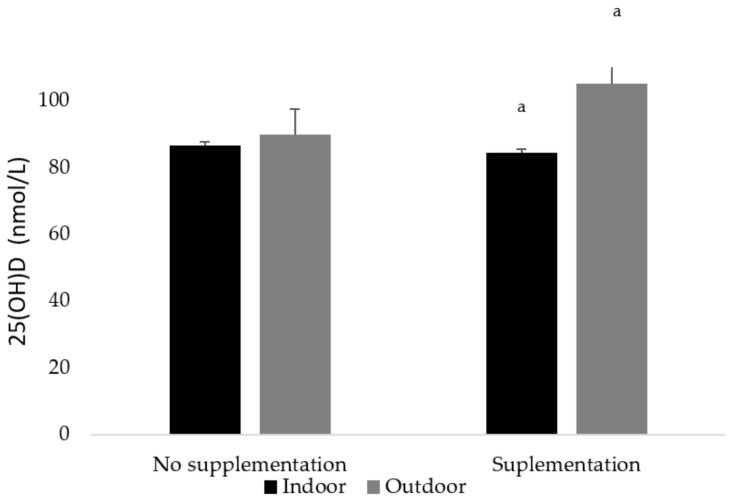
25(OH)D concentrations (nmol/L) according to training place and supplementation. ^a^ Significant differences were set up for outdoor supplemented players (*p* < 0.05).

Training environment had the highest influence on vitamin D concentrations (B = 0.296; *p* < 0.01) (data not shown). Moreover, a positive significant effect was observed for the interaction between indoor/outdoor training and supplementation on 25(OH)D concentrations (*p* < 0.05). Team players training outdoors and supplemented had higher total vitamin D concentrations (105 nmol/L) than those training indoors, irrespective of supplementation (84.6 nmol/L) (Figure 5).

## 4. Discussion

Novel results of vitamin D in elite players from six different first division sports were analyzed, considering several factors as the interaction between training environment and supplementation throughout the season, apart from anthropometric measures and skin color, with the aim to contribute to improving their global health and athletic performance. Some studies indicated a significant prevalence of vitamin D insufficiency in the general population worldwide [34,35]; nonetheless, limited comparable evidence is available about vitamin D status in elite team players and some of its determinants. Especially, there is a lack of information on vitamin D status, methodology, and supplementation and their interaction with the training environment [30].

A direct relationship has been demonstrated between serum 25(OH)D concentrations and muscle outcomes and bone mass [36]. The study of Koundourakis et al. (2014), performed in elite Greek, English, and Polish soccer players, suggested that vitamin D levels are related to muscle strength, sprinting capacity, and VO_2max_, irrespective of the levels of performance. They also found useful effects on vitamin D levels, proposing a probable relationship of vitamin D levels and training-induced stress [37].

Although Spain is a sunny country, our results indicate that approximately 30% of FCB team players were below optimal levels (<75 nmol/L) with seasonal variations. None of the studied team players were below the deficiency cut-off (<50 nmol/L) during the season. In previous studies performed in non-Spanish team players, deficiency prevalence ranged from 33.6% [5] up to 65% and 85% [7,9,38]. Specifically, in professional football players, in this case from the English Premier League, and contrary to our results, 65% of the sample were described as deficient (<50 nmol/L) in winter [39]. The lower latitude in Spain could protect our team players from a deficiency state, but not from insufficiency. Low vitamin D levels are frequently late onset rather than instant, assuming that people with vitamin D insufficiency are wholly asymptomatic, and it can make the diagnosis difficult [24]. This means that team players could experience decreased physical performance without relating it to vitamin D insufficiency. The first indication of vitamin D deficiency is muscle weakness, hypotonia, long time to peak muscle contraction, and muscle relaxation time [40]. In their study, Allison et al. (2014) concluded that 25(OH)D deficient players presented significantly slighter cardiac structural parameters than insufficient and optimal elite players [41].

Mean vitamin D concentrations of total FCB team players were above the optimal cut-off set at 75 nmol/L. Superior benefits have been observed at levels above 75 nmol/L. In our study, 39.5% (n = 42), 17.9% (19), and 17.9% (19) players maintain plasma 25(OH)D levels above 100 nmol/L in autumn and winter and spring, respectively (data are not shown). Strategies to ensure these vitamin D levels as a training environment or higher vitamin D intake should be considered. Vitamin D is mainly synthesized in the skin by casual exposure to ultraviolet B (UVB) sunlight and privation of sun exposure seems to be a risk issue for vitamin D insufficiency [42,43]. Ultraviolet light irradiation could increase physical fitness and decline chronic sports-related pain [19]. German Olympic officials considered these effects significant enough for UVB radiation to be contemplated as an ergogenic aid [19]. Regarding skin production, data show vitamin D synthesis might not compensate for low nutritional intakes [44]. Hypovitaminosis D can be found despite seemingly sufficient sun exposure [8,45], suggesting that a concomitant proper dietary intake is required. Important sources of vitamin D are oily fish such as, for example, salmon [3], irradiated mushrooms [4], and fortified food (milk, cheese, bread, among others). Vitamin supplementation could also play an important role in elite team players, as it is observed in the current study, and should be analyzed further.

The findings of this study suggest training outdoors has the highest influence on vitamin D concentrations. Results revealed that the highest mean vitamin D levels were observed in Caucasians training outdoors during the whole season, whereas the lowest were found in those training indoors, especially in basketball players, confirming previous data from our research group obtained from athletes from different sport modalities [11]. Furthermore, Maroon et al. (2015) found that Black football players had a greater risk of vitamin D deficiency than their White teammates [46]. Constantini et al. (2010) found vitamin D insufficiency levels among dancers (94%), basketball players (94%), and Taekwondo fighters (67%); nevertheless, they also obtained greater vitamin D deficiency prevalence among indoor training athletes compared to those training outdoors (80% vs. 48%; *p* < 0.01) [7]. A study of Sghaier-Ayadi et al. (2015) concluded that vitamin D status was significantly inferior in indoor than in outdoor players (36.2 ± 19.0 nmol/L vs. 49.1 ± 19.2 nmol/L; *p* < 0.001). Vitamin D deficiency (25-hydroxyvitamin D < 50 nmol/L) was linked to indoor training, with an odds ratio (95% CI) of 5.03 (1.64–15.4); *p* = 0.005) [47].

Our results are in accordance with studies across Europe regarding seasonality differences [44,48,49,50]. Mean vitamin D concentrations decreased as the season progressed. Higher mean values were obtained in autumn, after the sunny summer months (with 90% of the sample at optimal vitamin D concentrations), whereas lower levels were obtained in spring, after wintertime, with almost 40% of the sample at a vitamin D insufficiency state, especially in non-supplemented players. Constantini et al. (2010) also identified a greater prevalence of vitamin D insufficiency in athletes during winter months in a sunny country [7]. In the already mentioned study performed in elite soccer players of the English Premier League, a decrease in serum 25(OH)D levels was observed between August (104.4 ± 21.1 nmol/L) and December (51.0 ± 19.0 nmol/L) [40]. Supplementation was not included in all these analyzed studies [7].

Skin color is thought to be a key aspect that determines vitamin D concentration [51]. Dark-skinned people require more UV doses than light-skinned ones for analogous vitamin D biosynthesis [52]. We observed a trend for darker skin to be related to lower vitamin D concentrations, but only four dark skin players were analyzed. Therefore, the influence of skin color on 25(OH)D concentration in team players needs further studies. Likewise, the use of sunscreen could influence vitamin D mechanism deficiency [3], which requires further study.

In our study, the training environment had the highest influence on vitamin D concentrations and a significant and positive interaction of training place and supplement interaction on vitamin D status exists. Approximately 42% of our elite team players were supplemented from October to June. In general, those team players with supplementation had significantly higher vitamin D concentrations than those without it throughout the whole year. Football second division, fully supplemented, had the highest vitamin D levels, and supplemented team players managed to stay above optimal vitamin D concentrations during springtime.

On the contrary, 94.1% of handball players (also indoor training) were not supplemented. Their vitamin D concentrations suffered a consistent decrease from autumn to spring, reaching insufficiency levels. Supplementation could play an important role for maintaining their vitamin D levels around the season. Outdoor football players with supplementation had the highest vitamin D concentrations throughout the year. Among indoor modalities, indoor football and roller hockey were supplemented during the whole year, and despite their vitamin D levels, presented a decrease as the season progressed, with their vitamin D mean values staying above optimal vitamin D levels in springtime. The high vitamin D starting point in autumn of 118 nmol/L for indoor football and the full supplementation during the whole year could influence factors. On the contrary, basketball players, with a lower vitamin D starting point in autumn (77.8 nmol/L), could not keep their vitamin D concentration above optimal concentrations throughout the year, despite supplementation. Vitamin D levels at the start of the season, in autumn, seem to have an important role. Galán et al. (2012) projected 25(OH)D levels of >122.7 nmol/L in autumn to preserve optimal levels in spring. None of our team players reached this level. Our mean vitamin D concentrations in autumn were around 110 nmol/L, so higher levels in autumn should be desirable for the six sport modalities [27].

Most studies agree that cholecalciferol supplementation augmented total and free 25(OH)D concentrations consistently to the dose and without differences between races [53,54,55], but controversial data are still found about supplementation on physical performance. Vitamin D supplementation in individuals with low vitamin D status may improve muscle strength and have positive effects on muscular performance, decreasing the risk of injuries [56,57,58]. On the contrary, other studies concluded that supplementation had little impact on physical performance outcomes [55], even if it significantly improved vitamin D status in team players. However, the physiological vitamin D health effects of having adequate vitamin D levels remain. Vitamin D supplementation doses should also be analyzed in depth. Our supplemented team players took 5000 IU per week. In a recent study, Owens et al. (2017) concluded that high-dose vitamin D_3_ supplementation (70,000 IU·wk) could cause damage for its intended purposes because of increased 24,25(OH)_2_D production. Rapid withdrawal from high-dose supplementation may inhibit the bioactivity of 1,25(OH)2D_3_ because of sustained increases in 24,25(OH)_2_D that persist, as 25(OH)D and 1,25(OH)2D concentrations decrease [59]. Despite this, there is an ongoing discussion on the effect of vitamin D supplementation or fortified foods in young, healthy people [60]. The mixture between high vitamin D intake and UVB exposure is needed to achieve adequate vitamin D levels [61], especially in elite athletes, but adequate doses should be studied and considered.

The leading strength of our study was the presence of both a training environment and supplementation in observing vitamin D status in Spanish elite team players of one of the most important football clubs worldwide. Limited comparable information is available. Some studies have found vitamin D insufficiency in team players considering only outdoor training, but there is a lack of studies of vitamin D status and reliable methods. Moreover, first division Spanish team sport players have not been investigated yet. Our results contribute to a better understanding of vitamin D concentrations throughout the sport season. There are some limitations in our study. Vitamin D was not analyzed using the gold standard method, which is liquid chromatography-tandem mass spectrometry. Likewise, the authors were not available to analyze other vitamin D-related parameters such as, for example, PTH, calcium, or vitamin D-binding protein. Unfortunately, due to the difficulties in assessing elite team players and testing them, only limited data could be collected, and other unmeasured confounders could have influenced our observations.

## 5. Conclusions

Spanish team players show a risk of hypovitaminosis D. Season, skin color, outdoor training, and supplementation are the most important factors influencing vitamin D concentrations in elite team players. In fact, a positive interaction with supplementation exists in two different directions; outdoor training improves vitamin D status only in supplemented team players and supplementation has a positive influence on vitamin D status only in individuals with adequate sun exposure. Vitamin D levels at the beginning of the season, in autumn, seem to have an important role in maintaining adequate vitamin D levels throughout the sport season. This study provides an inside perspective of the connections between training environment and supplementation on vitamin D concentrations.

The results can contribute to identify those team players at vitamin D insufficiency risk, especially during winter and springtime. Vitamin D deficiency might affect team players’ overall health and performance; therefore, vitamin D status should be routinely screened in order that team players can be coached, especially during the winter season, to maintain serum 25(OH)D concentrations of ≥75 nmol/L and preferably ≥100 nmol/L in autumn. Regular safe sun exposure with outdoor training and/or dietary supplementation combined with increased vitamin D intake, especially in basketball or handball players, is recommended. Further longitudinal studies are desirable in elite teams.

## Figures and Tables

**Table 1:** Descriptive characteristics of the sample. Vitamin D concentrations (nmol/L) according to sufficient–insufficient cut-off points and training place for the total sample.

	%	Mean	SD	Min	Max
Age (y)	–	27.3	4.6	18.8	37.0
Height (cm)	–	184.7	11.1	167.0	218.0
Weight (kg)	–	82.5	12.4	64.0	120.0
Race	–				
Caucasian	87.4	93.6	23.2	52.6	159.8
Black	4.2	65.0	10.0	55.1	77.4
Other	8.4	85.1	17.2	69.8	118.4
Whole Mean 25(OH)D	–	91.9	23.1	52.6	159.8
<75 nmol/L	26.3	66.0	7.1	52.6	75.0
≥75 nmol/L	73.7	101.2	23.1	75.7	159.8
Autumn 25(OH)D status	–	109.8	32.8	60.8	220.3
<75 nmol/L	10	68.8	3.9	60.8	73.0
≥75 nmol/L	90	114.4	31.4	75.3	220.3
Winter 25(OH)D status	–	84.9	22.5	43.0	142.5
<75 nmol/L	34.6	61.8	8.7	43.0	73.8
≥75 nmol/L	65.4	97.2	17.3	75.8	142.5
Spring 25(OH)D status	–	82.2	21.8	31.8	138.3
<75 nmol/L	38.6	61.8	9.8	31.8	74.5
≥75 nmol/L	61.4	94.9	17.0	75.3	138.3
25(OH)D Indoor Training place	54.7	85.3	23.1	52.6	159.8
Handball	17.9	95.5	24.5	52.6	145.8
Basketball	12.6	67.7	9.9	53.1	83.5
Roller Hockey	11.6	76.4	10.8	62.2	93.25
Indoor Football	12.6	98.0	25.1	69.8	159.8
25(OH)D Outdoor Training place	45.3	99.5	21.0	58.0	141.2
Football 1st division	23.2	95.0	22.0	58.9	141.2
Football 2nd division	22.1	104.2	19.3	58.0	132.1
Supplementation (YES)	58.7	–	–	–	–

Sufficient cut-off point set up as >75 nmol/L and insufficient as <75 nmol/L. Statistically significant with *p* < 0.01.

**Table 2:** T-test between age, weight, height, and 25(OH)D concentrations (nmol/L) according to supplementation.

		N	Mean	SD	*p*-Value
**Age**	No supplementation	35	28.1	4.0	0.046
	Supplementation	60	26.9	4.9	
**Height**	No supplementation	35	187.2	11.8	NS
	Supplementation	60	182.6	10.6	
**Weight**	No supplementation	35	84.8	13.1	NS
	Supplementation	60	80.4	11.9	
**Autumn (mean 25(OH)D)**	No supplementation	39	107.7	33.2	NS
	Supplementation	56	111.2	32.8	
**Winter (mean 25(OH)D)**	No supplementation	39	87.4	22.8	NS
	Supplementation	56	82.7	22.0	
**Spring (mean 25(OH)D)**	No supplementation	39	73.6	16.8	0.014
	Supplementation	56	89.0	23.6	

NS: non-significant. Statistically significant results are in bold (*p* < 0.05).

**Table 3:** Descriptive mean values of vitamin D concentrations (nmol/L) as mean, SD, minimum, and maximum according to season, sport modality, and supplementation.

	Sports	Mean	SD	Min	Max	*p*-Value
**WHOLE** **YEAR**	**Handball**	95.5	24.5	52.6	145.8	
	No Supplementation	93.7	24.0	52.6	145.8	NS
	Supplementation	125.6	–	125.6	125.6	NS
	**Basketball (BS)**	67.7	9.9	53.1	83.5	<0.05 with all
	**Roller hockey (RH)**	76.4	10.8	62.2	93.25	<0.05 with football 2nd div
	**Indoor Football**	98.0	25.1	69.8	159.8	NS
	**Football 1st div**	95.0	22.0	58.9	141.2	NS
	No Supplementation	90.0	19.8	58.9	127.1	NS
	Supplementation	108.5	23.6	83.3	141.2	NS
	**Football 2nd div**	104.2	19.3	58.0	132.1	NS
**AUTUMN**	**Handball**	118.5	39.2	78.5	220.3	NS
	No Supplementation	117.9	40.4	78.5	220.3	NS
	Supplementation	127.5	–	127.5	127.5	NS
	**Basketball**	77.8	19.2	60.75	105.3	<0.05 with all except RH
	No Supplementation	99.0	20.5	70.0	137.0	NS
	Supplementation	132.7	26.6	105.0	158.5	NS
	**Roller hockey**	87.0	13.6	71.6	116.5	NS
	**Indoor Football**	115.9	39.5	65.5	210.5	NS
	**Football 1st div**	107.4	26.1	70	158.5	NS
	No Supplementation	99.0	20.5	70.0	137.0	<0.05 between suppl.
	Supplementation	132.7	26.6	105.0	158.5	NS
	**Football 2nd div**	120.6	30.0	68.7	168.2	NS
**WINTER**	**Handball**	95.7	25.8	43	142.5	NS
	No Supplementation	92.8	23.5	43.0	137.0	NS
	Supplementation	142.5	–	142.5	142.5	NS
	**Basketball**	71.5	11.7	55.3	92.3	<0.05 with all except RH
	No Supplementation	77.8	8.1	68.8	92.3	<0.05 between suppl.
	Supplementation	58.8	5.1	55.3	66.3	NS
	**Roller hockey**	63.7	11.1	50.0	89.7	<0.05 with all except BS
	**Indoor Football**	–	–	–	–	NS
	**Football 1st div**	90.7	23.6	54.5	130.5	NS
	No Supplementation	86.8	24.5	54.5	130.5	<0.05 between suppl.
	Supplementation	100.3	19.6	83.5	130.5	NS
	**Football 2nd div**	89.6	17.5	50.0	121.0	NS
**SPRING**	**Handball**	72.4	17.7	31.8	106.8	<0.01 with football 2nd div
	No Supplementation	70.3	15.9	31.8	94.8	NS
	Supplementation	106.8		106.8	106.8	NS
	**Basketball**	61.4	10.6	50.3	83.0	<0.01 with football (both)
	No Supplementation	71.6	14.9	54.8	83.0	<0.05 between suppl.
	Supplementation	57.9	6.8	50.3	70.0	NS
	**Roller hockey**	82.3	12.6	67.0	110.8	NS
	**Indoor Football**	80.2	16.7	51.5	109.25	<0.05 with football 2nd div
	**Football 1st div**	86.7	20.7	50.8	134.5	NS
	No Supplementation	81.1	17.1	50.8	114.3	<0.05 between suppl.
	Supplementation	100.8	23.7	77.8	134.5	NS
	**Football 2nd div**	103.6	22.3	55.3	138.3	NS

BS: basketball; RH: roller hockey. RH, indoor football, and football 2nd division were fully supplemented during the whole year (100% of the players). Handball (5.9%), basketball (33.0%), and football 1st division (27.3%) players were supplemented.

## Data Availability

The data presented in this study are available on request from the corresponding author. The data are not publicly available due to privacy participants.
